# Human fetal liver MSCs are more effective than adult bone marrow MSCs for their immunosuppressive, immunomodulatory, and Foxp3^+^ T reg induction capacity

**DOI:** 10.1186/s13287-021-02176-1

**Published:** 2021-02-17

**Authors:** Yi Yu, Alejandra Vargas Valderrama, Zhongchao Han, Georges Uzan, Sina Naserian, Estelle Oberlin

**Affiliations:** 1 State Key Laboratory of Experimental Hematology, Institute of Hematology & Blood Diseases Hospital, Chinese Academy of Medical Sciences & Peking Union Medical College, Tianjin, China; 2 INSERM UMR-S-MD 1197, Hôpital Paul Brousse, Villejuif, France; 3 Beijing Institute of Stem Cells, Health & Biotech Co., Ltd, Beijing, People’s Republic of China; 4 Paris-Saclay University, Villejuif, France; 5 CellMedEx, Saint Maur des Fossés, France

**Keywords:** Fetal liver, Adult bone marrow, Mesenchymal stem cells, T cell immunomodulation, Induced regulatory T cells

## Abstract

**Background:**

Mesenchymal stem cells (MSCs) exhibit active abilities to suppress or modulate deleterious immune responses by various molecular mechanisms. These cells are the subject of major translational efforts as cellular therapies for immune-related diseases and transplantations. Plenty of preclinical studies and clinical trials employing MSCs have shown promising safety and efficacy outcomes and also shed light on the modifications in the frequency and function of regulatory T cells (T regs). Nevertheless, the mechanisms underlying these observations are not well known. Direct cell contact, soluble factor production, and turning antigen-presenting cells into tolerogenic phenotypes, have been proposed to be among possible mechanisms by which MSCs produce an immunomodulatory environment for T reg expansion and activity. We and others demonstrated that adult bone marrow (BM)-MSCs suppress adaptive immune responses directly by inhibiting the proliferation of CD4^+^ helper and CD8^+^ cytotoxic T cells but also indirectly through the induction of T regs. In parallel, we demonstrated that fetal liver (FL)-MSCs demonstrates much longer-lasting immunomodulatory properties compared to BM-MSCs, by inhibiting directly the proliferation and activation of CD4^+^ and CD8^+^ T cells. Therefore, we investigated if FL-MSCs exert their strong immunosuppressive effect also indirectly through induction of T regs.

**Methods:**

MSCs were obtained from FL and adult BM and characterized according to their surface antigen expression, their multilineage differentiation, and their proliferation potential. Using different in vitro combinations, we performed co-cultures of FL- or BM-MSCs and murine CD3^+^CD25^−^T cells to investigate immunosuppressive effects of MSCs on T cells and to quantify their capacity to induce functional T regs.

**Results:**

We demonstrated that although both types of MSC display similar cell surface phenotypic profile and differentiation capacity, FL-MSCs have significantly higher proliferative capacity and ability to suppress both CD4^+^ and CD8^+^ murine T cell proliferation and to modulate them towards less active phenotypes than adult BM-MSCs. Moreover, their substantial suppressive effect was associated with an outstanding increase of functional CD4^+^CD25^+^Foxp3^+^ T regs compared to BM-MSCs.

**Conclusions:**

These results highlight the immunosuppressive activity of FL-MSCs on T cells and show for the first time that one of the main immunoregulatory mechanisms of FL-MSCs passes through active and functional T reg induction.

**Supplementary Information:**

The online version contains supplementary material available at 10.1186/s13287-021-02176-1.

## Background

Mesenchymal stem cells (MSCs) have been first identified by Friedenstein et al. [1]. They are multipotent stromal cells that can self-renew and differentiate into various mesodermal cell types including chondrocytes, osteocytes, and adipocytes [2, 3]. They can also differentiate into cell types of other germ layers, like neurons (ectoderm) and hepatocytes (endoderm) [4, 5]. The first MSC isolations were made from bone marrow (BM) samples based on the plastic adherence property of these cells as opposed to the hematopoietic cells which remained in suspension [6]. Since, MSCs have been isolated from numerous other organs/tissues in adult [7, 8], neonatal [9, 10], and fetal-stage tissues [11–13].

MSCs represent one of the most promising stem cells for regenerative medicine due to their multipotency, their low immunogenicity, their ease of isolation from multiple accessible tissues, and their adaptability to large scale ex vivo culture expansion [14–16]. Beside their regenerative features, MSCs have substantial immunosuppressive potential [17–21]. Regarding this property, MSCs have been shown to play a role in down-modulating the innate immune response, by interfering the maturation and antigen-presenting capacity of dendritic cells [22, 23], and reducing the proliferation and cytotoxicity of natural killer (NK) cells [24–26]. MSCs also dampen the adaptive immune response, by modulating B cell functions [27] and by suppressing both CD4^+^ and CD8^+^ T cell functions [28–30]. An additional mechanism by which MSCs may suppress antigen-specific T cell responses is indirectly via regulatory T cell (T reg) induction [31, 32]. In 2008, Di Ianni et al. revealed an enhanced frequency of T reg production and long-lasting T reg immunosuppressive activities when T cell subpopulations were co-cultured with MSCs [33]. Experimental studies including ours have since been published to confirm this phenomenon and shed new light on mechanistic aspects [34–40]. Furthermore, ex vivo expanded MSCs exhibit effective immunomodulatory effects in a wide range of animal experimental models and was validated in several clinical trials as safe, feasible, and potent immunotherapies for human immune-mediated disorders including graft-versus-host disease (GVHD) and autoimmune diseases [41–44]. A large number of these animal model studies [45–50] and clinical trials [51–58] have also documented changes in T reg number and function after systemic or localized administration of either autologous or allogenic MSCs.

However, to date, most data on immunosuppressive properties of MSCs is based on studies with BM-MSCs rather than neonatal or fetal MSCs. Although there are fewer studies emerging from these later sources, umbilical cord blood, umbilical cord (Wharton’s jelly), placenta membranes (amnion and chorion), amniotic fluid, and fetal liver (FL)-MSCs have been shown to represent an intermediate between embryonic and adult cells, regarding proliferation rates, plasticity features, and immunomodulatory properties [59–62]. Among these alternative sources of MSCs, FL-MSCs seem to be one of the most promising [63, 64]. We and others demonstrated that FL-MSCs display higher proliferative capacities and much longer-lasting immunomodulatory properties compared to BM-MSCs, by inhibiting directly the proliferation and activation of NK cells and also those of CD4^+^ and CD8^+^ T cells [65–67]. Notably, no information is currently available about the capacity of FL-MSCs to induce functional T regs from conventional T cells (T convs). As fetal secondary lymphoid organs exhibited an higher frequency of T regs as compared to any other time during life [68–71] and since fetal naïve CD4^+^ T cells display an outstanding capacity to convert into T regs when stimulated with alloantigens, as compared to adult naïve CD4^+^ T cells [72, 73], it seemed important to us to check if FL-MSCs could exert their immunosuppressive effect also indirectly through induction of T regs.

We have thus isolated and compared MSCs from FL and adult BM sources to gain additional insight into their phenotype, differentiation, and proliferative potential. We compared these cells in order to identify if they display significant differences in their immunosuppressive efficacy, either directly on CD3/CD28-stimulated CD3^+^CD25^−^ murine T convs or indirectly through T reg induction.

Although both types of MSCs demonstrated similar phenotypic profiles and differentiation capacity, FL-MSCs displayed greater ex vivo expansion ability than adult BM-MSCs. Furthermore, after co-culturing with CD3/CD28-stimulated CD3^+^CD25^−^ murine responder T cells, FL-MSCs decreased much more T cell proliferation and modulated them towards less active phenotypes than adult BM-MSCs. In addition to their substantial suppressive effect, we have revealed for the first time that FL-MSCs more effectively promoted the transformation of CD3/CD28-activated CD3^+^CD25^−^ murine T convs into active and functional CD4^+^CD25^+^ Foxp3^+^ T regs. Because of their outstanding proliferative capacities and much stronger in vitro immunosuppressive properties, FL-MSCs could be considered as a new and efficient source for MSC therapy of immune-mediated/inflammatory diseases or to prevent allograft rejection.

## Methods

### Human tissues

Human fetal livers (FL) (range 7–9 weeks of gestation) were obtained from voluntary abortions (Obstetrics and Gynecology Department, Rene Dubos Hospital, Pontoise, France). Developmental age was estimated based on several anatomic criteria according to the Carnegie classification for embryonic stages [74] and by ultrasonic measurements for fetal stages. Bone marrow (BM) aspirates were obtained from patients undergoing a total hip replacement surgery (HIA, Percy Hospital, Clamart, France and Orthopedic Service, Polyclinic of Blois, Blois, France). Human adult peripheral blood from healthy adults was obtained from the French Establishment of Blood (EFS) (Rungis, France).

### MSC isolation and culture

FL samples were excised sterilely using microsurgery instruments and a dissecting microscope, in phosphate-buffered saline (PBS) containing 1% penicillin/streptomycin (P/S) (all from GIBCO). Tissues were dissociated for 1 h at 37 °C in α-minimum essential medium (α-MEM; GIBCO) containing 10% fetal calf serum (FCS; Eurobio), 0.1% type I/II/IV collagenase, and type VIII hyaluronidase (Sigma-Aldrich). FLs were then disrupted mechanically through 18-, 23- and 26-gauge needles successively. BM cells were extracted and disrupted mechanically from hip surgery samples, in PBS containing 1% P/S (all from GIBCO). BM and FL mononuclear cells were then isolated using a density Ficoll gradient separation (Pan-Biotech) and plated at 1.5 × 10^5^ cells/cm^2^ in α-MEM medium supplemented with 1% GlutaMAX, 1% P/S solution (all from GIBCO), and 10% heat-inactivated FCS (Eurobio) at 37 °C in 5% CO_2_. After 3 days, non-adherent cells were discarded and the medium was changed every week. When the adherent cells were 70–80% confluent, they were harvested by treating with trypsin-EDTA (GIBCO) and seeded at 4000/cm^2^ in α MEM complete medium. MSCs were amplified from passages 0 to 3. In all experiments, MSCs were used from passage 3 to 6. For morphology analysis images were acquired at × 4 magnification at early, mid, and late passage numbers using the EVO XL Core Imaging System from Life technologies. For immune-phenotyping analysis, MSCs were recovered and stained with fluorochrome-conjugated antibodies for MSCs (APC-anti CD271 (LNGFR), PE-anti-CD166 (ALCAM), APC-anti-CD146 (MCAM), FITC-anti-CD90 (Thy-1), PE-anti-CD106 (VCAM-1), PE-anti-CD105 (Endoglin), PE-anti-CD73, APC-anti-CD54 (ICAM-1), APC-anti-CD51 (Integrin αV), PE-anti-CD44 (HCAM), APC-anti-CD29 (β1-integrin)), hematopoietic (FITC-anti-CD45, PE-anti-CD14, and APC-anti-CD34), and endothelial (PE-anti-CD144) markers and major histocompatibility (MHC) (FITC-anti-HLA-DR, FITC-anti-HLA ABC) antigens as described below.

### MSC differentiation assay

FL and BM-MSCs were tested for their ability to differentiate into adipocytes and osteocytes (R&D Systems). For osteogenic differentiation, MSCs were seeded in 24-well plates at 3000/cm^2^ in α-MEM supplemented with 10% FCS and 1% P/S. After cell adhesion, medium was removed and replaced by α-MEM supplemented with 10% FCS, 1% P/S and 0.052 μg/ml dexamethasone, 12.8 μg/ml ascorbic acid, and 2.15 mg/ml β-glycerophosphate from Sigma-Aldrich. Cells were cultured for 12 days to 3 weeks at 37 °C in 5% CO_2_ atmosphere, and medium was changed 2 times in a week. Quantification of mineralization was performed after fixation in 4% paraformaldehyde for 10 min and incubation in a solution of 2% Alizarin Red S (Sigma-Aldrich) for 5 min. Cells were then washed and dried. A buffer composed of 0.5 N hydrochloric acid and 5% SDS was added for extraction of Alizarin Red S staining and read at 405 nm. For adipogenic differentiation, MSCs were seeded at 21,000/cm^2^ in α-MEM medium supplemented with 10% FCS and 1% antibiotics. After cells reached confluence, medium was removed and DMEM high glucose supplemented with 10% FCS, 1% antibiotics, 0.52 μg/mL dexamethasone, 0.2 mM indomethacine, 0.01 mg/mL insulin, and 0.5 mM 3-isobutyl-1-methylxanthine (IBMX) (All from Sigma-Aldrich) were added for 3 days. Medium was removed and DMEM high glucose supplemented with 10% FCS, 1% antibiotics, and 0.01 mg/mL insulin were added for 1 day. Medium was removed for 2 additional cycles as previously and incubated in DMEM high glucose supplemented with 10% FCS, 1% antibiotics, and 0.01 mg/mL insulin for the last week. Cells were cultured for 3 weeks at 37 °C in 5% CO_2_ atmosphere. Differentiation into adipocytes was evaluated after fixation in 4% paraformaldehyde for 10 min and incubation in a solution of 0.3% Oil Red O for 5 min.

### MSC proliferation assay

FL and BM-MSC proliferation potential was evaluated using the CellTiter 96® AQueous Non-Radioactive Cell Proliferation Assay (Cat. N^o^ G3582, Promega) according to the manufacturer’s instruction. The amount of soluble formazan product produced by the reduction of MTS by metabolically active cells was measured at the 490 nm absorbance using the microplate fluorometer Fluoroskan Ascent® from Thermo Fisher Scientific.

### T cell isolation and culture

Murine pan T cell isolation kit II (Miltenyi Biotec) was used to isolate total CD3^+^ T cells from pooled spleens of 6 to 12 weeks old WT C57BL/6 mice (Envigo and Charles River).

Human pan T cell isolation kit (Miltenyi Biotec) was used to isolate total CD3^+^ T cells from MNCs of peripheral adult blood. Furthermore, CD25^+^ cells were depleted from the mouse and the human CD3^+^ T cell population using either anti-mouse CD25 biotin-conjugated antibody (BD biosciences) or anti-human CD25 biotin-conjugated antibody (Miltenyi Biotec), and anti-biotin microbeads (Miltenyi Biotec). The magnetic-activated cell sorting (MACS) method was used in all cell isolation steps. The resulting murine or human CD3^+^CD25^−^ T cells, ≥ 95% pure, were cultured in the presence of BM and FL-MSCs.

### MSCs/T cells co-culture

5 × 10^4^ BM- or FL-MSCs were seeded in 6-well plates and incubated for 24 h in α-MEM containing low glucose, 1% GlutaMAX, 1% P/S (all from Gibco), and 10% heat-inactivated FCS (Eurobio). Freshly isolated murine CD3^+^CD25^−^T cells were then added at different ratios to BM- or FL-MSCs, depending on experimental conditions, in RPMI medium containing 1% P/S, 1% HEPES, 5 × 10^− 5^ M β-mercaptoethanol (all from GIBCO), and 10% FCS (FCS; Eurobio). All co-cultures were performed in 50% α-MEM-50% RMPI complete medium at 37 °C in 5% CO_2_. For FACS analysis, T cells were removed by a gentle cell re-suspension followed by a cell aspiration (T cells stay in suspension and MSCs adhere to the plastic). In order to avoid the integration of potentially contaminating MSCs to T cell results, cells were first gated on CD4^+^ or CD8^+^ T cell markers prior analyzing any further markers.

### T lymphocytes proliferation assay

Freshly isolated CD3^+^CD25^−^ murine or human T cells were labeled with carboxy fluorescein diacetate succinimidyl ester (CFSE) (Molecular Probes) and stimulated by Dynabeads mouse or human T-activator CD3/CD28 respectively (Gibco) according to the supplier’s protocol. 5 × 10^4^ BM- or FL-MSCs seeded in 6-well plates 1 day before were then co-cultured with increasing numbers of CFSE-labeled, activated CD3^+^CD25^−^ murine, or human responder T cells (MSC to T cell ratios used 1:1, 1:2, 1:4, 1:6, 1:8, and 1:10) in a total volume of 3 ml of 50% RPMI-50% MEMα medium. 1 × 10^5^ CFSE-labeled, activated or non-activated murine or human CD3^+^CD25^−^ T cells grown alone in 50% RPMI-50% MEMα medium were used as controls. After 3 days of co-culture, murine or human T cells were harvested by gentle aspiration and stained with Vioblue-anti-mouse CD4 and PE-Vio770-anti-mouse CD8α antibodies or Vioblue-anti-human CD4 and PE-Vio770-anti-human CD8 antibodies, respectively (Miltenyi Biotec). The percentage of proliferating cells among CD4^+^ and CD8^+^ T cells was analyzed by flow cytometry measurements of the dilution of CFSE using LSRFortessa flow cytometer (BD Biosciences) and the FlowJo v10 software’s proliferation tool (FlowJo LLC). Cells undergoing division were identified by the decrease in CFSE resulting from dilution of the dye with each division. CFSE-labeled, non-activated murine CD3^+^CD25^−^ T cells grown alone consisted of non-proliferating cells (CFSE bright) with less than 5% CFSE dim proliferating cells.

### T lymphocytes activation assay

Freshly isolated CD3^+^CD25^−^ murine T cells were stimulated by Dynabeads mouse T-activator CD3/CD28 (Gibco) according to the supplier’s protocol. 5 × 10^4^ BM- or FL-MSCs seeded in 6-well plates 1 day before were then co-cultured with 25 × 10^5^ activated CD3^+^CD25^−^ murine responder T cells (MSC to T cell ratio used 1:5) in a total volume of 3 ml of 50% RPMI-50% MEMα media. 1 × 10^5^ activated and non-activated murine CD3^+^CD25^−^ T cells grown alone in 50% RPMI-50% MEMα medium were used as controls. After either 1 or 3 days, murine T cells were harvested by gentle aspiration and stained with either VIOBLUE-anti-CD4, VioBright FITC-anti-CD8α, PE-anti-GITR, PE-Cy7-anti-CD25 (all from Miltenyi Biotec) and PE-Cy5.5-anti-Foxp3 (eBioscience) or anti-CD4-VIOBLUE, FITC-anti-CD8α, PE-Vio770-anti-ICOS, APC-anti-TNFR2 (all from Miltenyi Biotec), and PE-Cy5.5-anti-Foxp3 (eBioscience) antibodies (Abs).

### Regulatory T cell induction assay

Freshly isolated CD3^+^CD25^−^ murine T cells were stimulated by Dynabeads mouse T-activator CD3/CD28 (Gibco) according to the supplier’s protocol. 5 × 10^4^ BM- or FL-MSCs seeded in 6-well plates 1 day before were then co-cultured with increasing numbers of activated murine CD3^+^CD25^−^ responder T cells (MSC to T cell ratios used 1:1, 1:2, 1:4, 1: 6, 1:8, and 1:10) in a total volume of 3 ml 50% RPMI-50% MEMα medium. 1 × 10^5^ murine activated and non-activated CD3^+^CD25^−^ murine T cells grown alone in culture were used as controls. After 4 days, T cells are harvested and stained using the following Abs: VIOBLUE-anti-CD4, FITC-anti-CD8α, PE-Cy7-anti-CD25, PE-anti-CTLA4, APC-anti-TNFR2 (all from Miltenyi) and Foxp3-PE-Cy5.5 (eBioscience) or anti-CD4-VIOBLUE, FITC-anti-CD8α, PE-anti-GITR, PE-Vio770-anti-ICOS (all from Miltenyi Biotec), and PE-Cy5.5-anti-Foxp3 (eBioscience).

### Induced T reg suppressive capacity assay

The CD4^+^CD25^+^ regulatory T cell isolation kit (Miltenyi Biotec) was used to isolate induced regulatory T cells (iT regs) following a 4-day co-culture of activated CD3^+^CD25^−^ murine T cells with FL- or BM-MSCs. Briefly, CD4^+^ T cells were first negatively selected on a separation column; then, the CD4^+^CD25^+^ subset was purified using CD25 microbeads (Miltenyi Biotech) according to the manufacturer’s instructions. To determine the purity of the isolated iT regs, we performed a flow cytometry analysis using VIOBLUE-anti-CD4, FITC-anti-CD8α, PE-Cy7-anti-CD25 (all from Miltenyi), and Foxp3-PE-Cy5.5 (eBioscience) antibodies combination. The purity of CD4^+^CD25^+^ iT regs was consistently higher than 90%, as confirmed by FACS. Isolated iT regs were then co-cultured in 96-well round bottom plates with increasing number of freshly isolated, CSFE-labeled and CD3/CD28-activated murine CD3^+^CD25^−^ responder T cells using different iT reg to T cell ratios (1:1, 1:5, 1:10) in a total volume of 200 μl of RPMI medium containing 1% P/S, 1% HEPES, 5 × 10^− 5^ M β-mercaptoethanol (all from GIBCO), and 10% FBS (Pansera ES; PAN-Biotech) at 37 °C in 5% CO2 as previously described [75]. 1 × 10^5^ CSFE-labeled, activated and non-activated murine CD3^+^CD25^−^ T cells grown alone in culture were used as controls. After 3 days of co-culture, cells were harvested and stained using VIOBLUE-anti-CD4 and FITC-anti-CD8α antibodies combination. The percentage of proliferating cells among CD4^+^ and CD8^+^ T cells was analyzed by flow cytometry measurements of the dilution of CFSE using LSRFortessa flow cytometer (BD Biosciences) and the FlowJo v10 software’s proliferation tool (FlowJo LLC). CFSE-labeled, non-activated T cells cultured alone were used as non-proliferating cell control (CFSE bright) with less than 5% CFSE dim proliferating cells. CSFE-negative cells corresponding to iT regs were gated out for the analysis. To ensure that only T conv cell proliferation was measured and that iT regs did not contribute to the proliferation, observed wells containing only iT regs were included in all experiments.

### MSC and T cell cell analysis by flow cytometry

Antibodies used for flow cytometry analysis are listed in Supplementary Table 1.

For MSC immune-phenotyping, cells developed in culture were harvested by non-enzymatic treatment (Cell dissociation solution; Sigma-Aldrich) and washed in complete medium and re-suspended in PBS (GIBCO) 0.2% bovine serum albumin (BSA) (Sigma-Aldrich). For T cells immune-phenotyping, cells were recovered by gentle aspiration, washed in complete medium and washed and re-suspended in PBS (GIBCO) 0.2% bovine serum albumin (BSA) (Sigma-Aldrich). In all cases, 10^5^ recovered cells were incubated for 30 min on ice with fluorochrome-conjugated antibodies, washed and re-suspended in PBS (GIBCO) 0.2% bovine serum albumin (BSA) (Sigma-Aldrich), for analysis.

Intracellular Foxp3 staining was performed after cell surface antibodies staining, according to the manufacturer’s instructions, using Foxp3 staining buffer set from eBioscience. Flow cytometric analysis was performed using LSRFORTESSA flow cytometer (BD Biosciences) and analyzed using FlowJo v10 software (FlowJo LLC). Background staining was evaluated using isotype-matched control antibodies and 7-Amino-Actinomycine D (7AAD) (Sigma-Aldrich) was used to gate dead cells out.

### Statistical analysis

Prism software (GraphPad) was used for statistical analysis. Student *t* test or one-way ANOVA with post hoc analysis was performed depending on the number of comparatives. For cytometry analysis, we have normalized the mean fluorescence intensity (MFI) values with T cell alone control group or BM-MSC-derived iT regs for T regs activation marker experiments. Then, we used unpaired, two-tailed Student *t* tests or one-way ANOVA for *P* value generation. The data are represented as mean ± SEM. ns indicates non-significant; *P < .05, **P < .01, and ***P < .001. Correlation coefficient is significant at 0.8 < CC < 1 P***, 0.8 < CC < 0.6 P**, and 0.6 < CC < 0.4 P* and non-significant at CC > 0.4.

## Results

### FL-MSCs share several common characteristics with adult BM-MSCs and reveal a greater ex vivo expansion ability than adult BM-MSCs

**Fig. 1:**
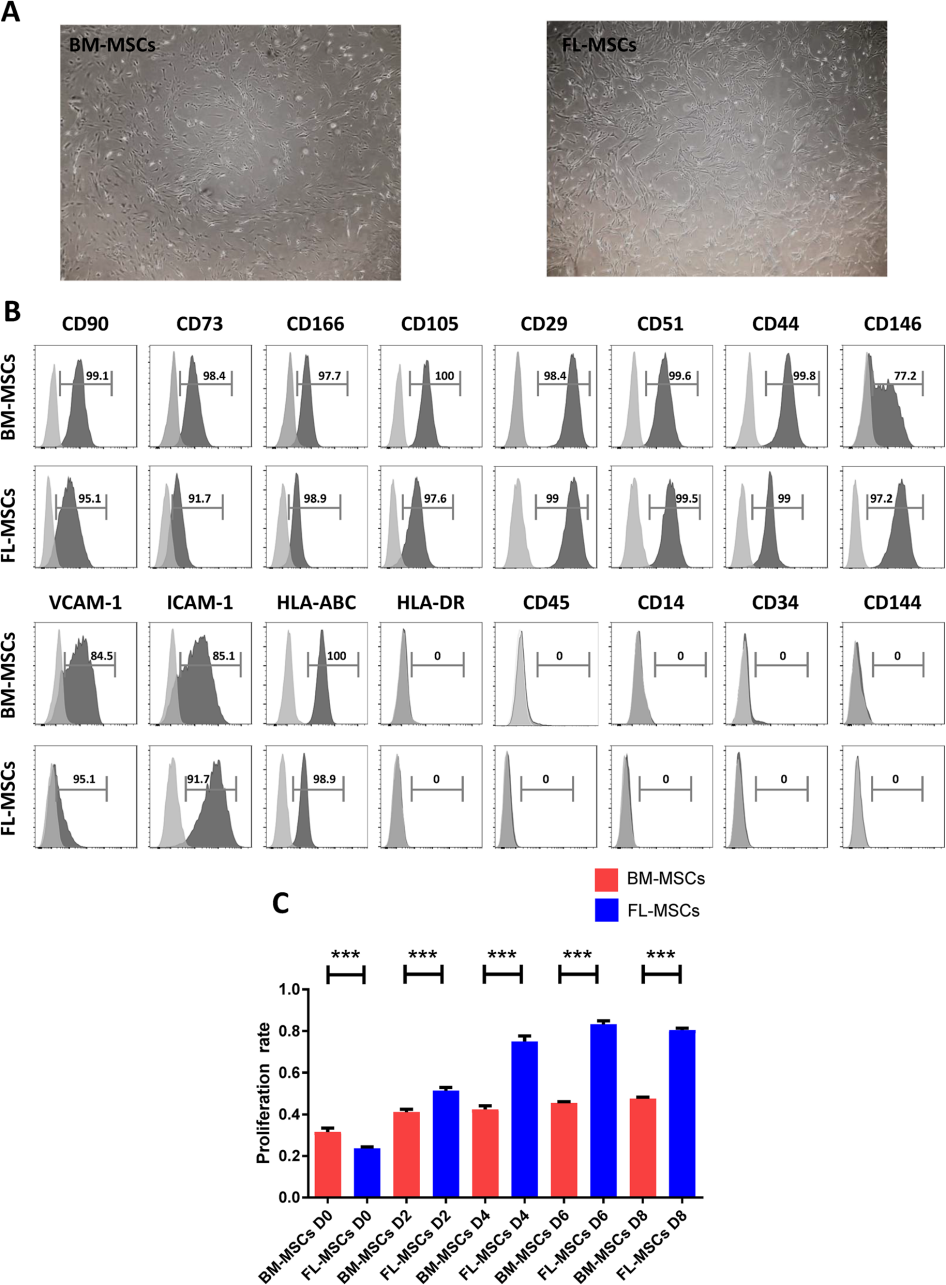
FL-MSCs share several common characteristics with adult BM-MSCs and reveal a greater ex vivo expansion ability than BM-MSCs. FL and BM-MSCs cultured between passage 3 and 6 were characterized by their cell morphology, their cell surface marker expression, and their differentiation and proliferative capacity. **a** After 14 days of culture, both FL (passage 3) and BM (passage 3)-MSCs showed a spindle-shaped fibroblast-like morphology representative of MSCS (original magnification × 4). Pictures are representative of 3 independent experiments (*n* = 3). **b** Flow cytometry analysis showing relative expression of MSC (CD271, CD166, CD146, CD90, CD106, CD105, CD73, CD54, CD51, CD44, CD29), hematopoietic (CD45, CD14, and CD34), and MHC (HLA-DR and HLA ABC) markers in FL (passage 3) and BM (passage 3)-MSCs. The dark gray histograms represent specific antibodies and light gray histograms represent corresponding isotype controls. Data are representative of 3 independent experiments (*n* = 6). **c** Comparison of proliferation of FL and BM-MSCs by MTS assay over a period of 8 days. Data are represented as mean value ± SEM from 3 independent experiments (*n* = 9). Unpaired Student t test was performed to generate *P* values. ns, non-significant; D, day. *P < .05, **P < .01, ***P < .001, ****P < .0001

Cells isolated from FL- or BM tissues were characterized by assessing their in vitro adherence, cell surface marker expression, lineage differentiation, and proliferation capacity. After 14 days of culture, both adult BM and FL-MSCs displayed a homogeneous spindle-shaped fibroblast-like morphology representative of MSCs (Fig. 1a). They were defined positive for human MSC characteristic markers such as CD271 (LNGFR), CD166 (ALCAM), CD146 (MCAM), CD90, CD106 (VCAM-1), CD105 (Endoglin), CD73, CD54 (ICAM-1), CD51, CD44, and CD29, and negative for hematopoietic (CD45, CD14, CD34) and endothelial (CD144) markers (Fig. 1b). They expressed human leukocyte antigen (HLA) class I molecules, but not HLA class II antigens (Fig. 1b). Even if both cell types express typical MSC markers, the MFI of some markers was extremely different. FL-MSCs had a higher expression of ICAM-1 and CD146 while expression of VCAM-1 was increased in BM-MSCs. FL-MSCs contained two populations of CD271^bright^ (which was demonstrated to have a much higher clonogenic capacity than CD271 low) [76–78], and CD271^low^ cells, while adult BM cultures contained only CD271^low^ cells (Supplementary Figure 1). Both FL and adult BM-MSCs had the ability to differentiate into adipocytes and osteocytes (Supplementary Figure 2). Cell viability and proliferation, quantified by the MTS assay over a period of 8 days, were significantly different between FL and BM-MSCs. Over the course of 8 days, FL-MSCs proliferated significantly more than BM-MSCs. Additionally, cells from BM and FL did not proliferate equally over the same time period. There was an increase in proliferation for BM-MSCs only in the 2 beginning days while FL-MSCs were proliferating progressively until 8 day, showing that FL-MSCs proliferate faster and for a longer period of time than adult MSCs (Fig. 1c).

### FL-MSCs exert more immunosuppressive function than BM-MSCs towards conventional T cells

**Fig. 2:**
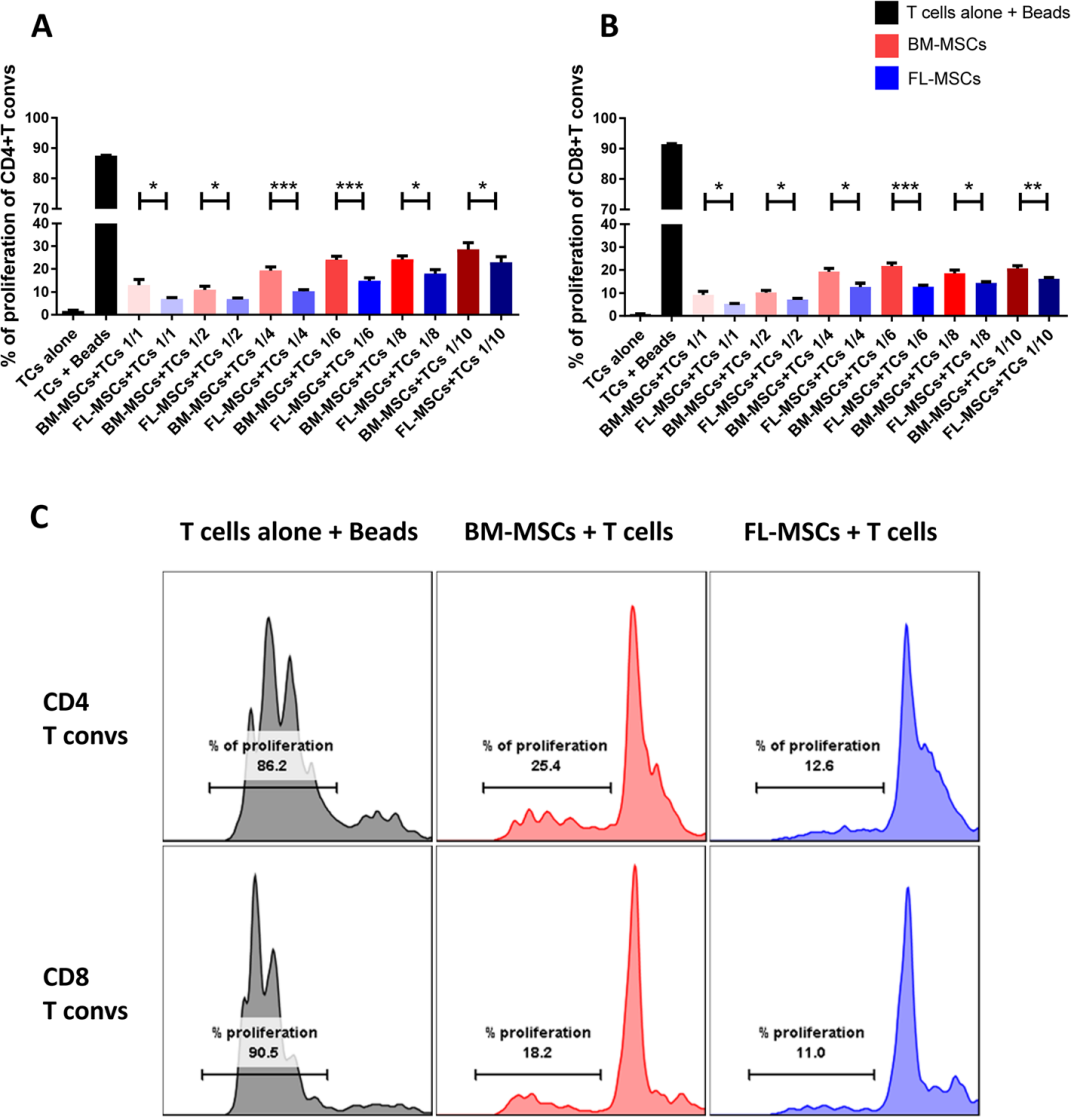
FL-MSCs are more immunosuppressive than BM-MSCs. CFSE-labeled, CD3/CD28-activated CD3^+^CD25^−^ effector T cells were co-cultured with FL-MSCs or BM-MSCs in 6 different MSC to T cell ratios. After 3 days, proliferation of CD4^+^ (**a**) and CD8^+^ T cells (**b**) was measured by flow cytometry based on CFSE dilution. Each histogram bar represents the percent of dividing cells. The first bar represents the unstimulated T cells alone (*n* = 6), the second bar represents the CD3/CD28-stimulated T cells alone (*n* = 6). Further bars depict T cells co-cultured with either BM-MSCs in red (*n* = 6) or FL-MSCs in blue (*n* = 6). All data are collected from 2 different experiments. Data are represented as mean value ± SEM. One-way ANOVA analysis was performed to generate *P* values. ns, non-significant; *P < .05, **P < .01, ***P < .001, ****P < .0001. **c** A flow cytometry representative of proliferation assay at 1:6 MSC to T cell ratio. T cells co-cultured with BM-MSCs are depicted in red and their FL counterparts are depicted in blue. Each histogram bar represents the percent of dividing cells. Beads, anti-CD3 and anti-CD28 activation beads; TCs, T cells; T convs, conventional T cells

MSCs are able to hinder T cell proliferation in response to allogenic and polyclonal CD3/CD28 stimulation. Here, we investigated if the origin of MSCs either from fetal or adult tissues can alter the ability of MSCs to suppress T cell proliferation. FL- or BM-MSCs were co-cultured with activated CD3^+^CD25^−^ murine T cells which were CFSE labeled, in 6 different MSC/T cells ratios. CD25^+^cells were removed from the primary pool of T cell population to avoid non-specific immunosuppression by CD25^+^ T regs [79]. Three days after, we collected T cells, cells in suspension, and the proliferation capacity of both T helper (CD4^+^) and cytotoxic T cells (CD8^+^) subpopulations was evaluated. T cells and MSCs require different appropriate cell culture media (RPMI and MEMα, respectively); therefore, we used a 1:1 mix in co-culture conditions. No difference was observed between T cells cultured in 100% RPMI or in 50% MEMα-50% RPMI (data not shown). In all examined ratios, CD4^+^CD25^−^ (Fig. 2a) and CD8^+^CD25^−^ (Fig. 2b) T cells co-cultured with MSCs (regardless of their origin) proliferated less than activated T cells alone (TCs + beads). However, we observed that FL-MSCs demonstrated a significantly more immunosuppressive effect than BM-MSCs towards both CD4^+^ and CD8^+^ T cells proliferation (Fig. 2a–c). This remarkable difference between BM and FL-MSCs was obvious starting from 1:1 ratio for both CD4^+^T cells (13.03% and 6.86% of proliferation, respectively) and CD8^+^ T cells (9.17% and 5.2% of proliferation, respectively). This difference was maintained until 1:10 ratio for CD4^+^T cells (28.58% and 22.88% of proliferation, respectively) and for CD8^+^T cells (20.65% and 16% of proliferation, respectively). These results clearly demonstrate the more powerful dose-dependent immunosuppressive effect of FL-MSCs compared to BM-MSCs towards T cells.

### FL-MSCs are stronger immune-modulators of conventional T cells compared to BM-MSCs

**Fig. 3:**
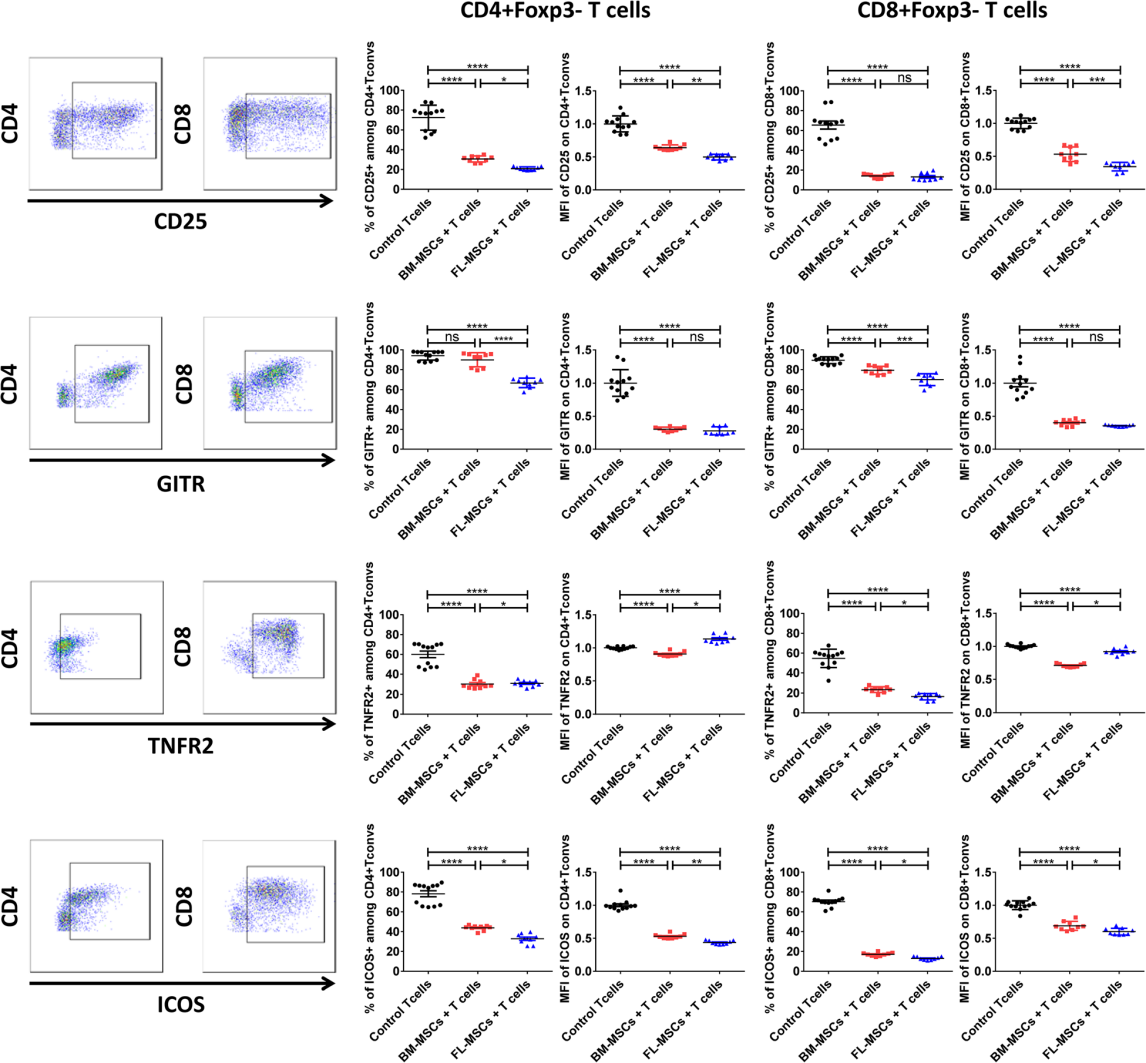
FL-MSCs down-modulate CD4^+^ and CD8+ T convs stronger than BM-MSCs. CD3/CD28 activated CD3^+^CD25^−^ effector T cells were co-cultured with BM-MSCs or FL-MSCs in a fixed 1:5 MSC to T cell ratio. After 1 day, T cells were collected and activation markers (CD25, GITR, TNFR2, and ICOS) were analyzed by flow cytometry. Representative flow cytometry dot plots showing the percentage of CD25, GITR, TNFR2, and ICOS, among CD4^+^Foxp3^−^ and CD8^+^Foxp3^−^T cells from the activated T cells control group (left panel). After delimitating the lymphocyte region by a forward-scatter-area (FSC-A) versus side-scatter area (SSC-A) plot, a CD4 versus Foxp3 plot and a CD8 versus Foxp3 plot were used to gate on CD4^+^Foxp3^−^T convs and CD8^+^Foxp3^−^ T cells, respectively. Frames defined the positive subpopulations for each marker analysis in the CD4^+^Foxp3^−^ and CD8^+^Foxp3^−^ populations. Statistical summary dot-plot graphs showing the percentage or the MFI value of each marker analyzed in CD4^+^Foxp3^−^and CD8^+^Foxp3^−^ T convs (right panel). Each dot represents a measured value collected from 2 different experiments (*n* = 12 for T cells + beads (black) and *n* = 9 for BM-MSCs + T cells (red) and FL-MSCs + T cells (blue) groups). For each group of values, horizontal lines represent mean value ± SEM. MFI values have been normalized with T cells + beads control group. One-way ANOVA analysis was performed to generate *P* values. ns, non-significant; Beads, anti-CD3 and anti-CD28 activation beads; T convs, conventional T cells. *P < .05, **P < .01, ***P < .001, ****P < .0001

Having observed more powerful immunosuppression by FL-MSCs, we investigated if these MSCs exert stronger immunomodulatory effect against T convs. FL and BM-MSCs were co-cultured with anti-CD3/CD28 activated murine CD3^+^CD25^−^T cells at a fixed 1:5 MSC to T cell ratio. After 1 day, T cells were harvested and analyzed for the expression of different activation markers upregulated by activated CD4^+^Foxp3^−^ (CD4 T convs) or CD8^+^Foxp3^−^ (CD8 T convs) T cells. At first, the expression of CD25, the α-chain of the IL-2 receptor, was quantified. We observed a dramatic decrease of the percentage of CD25^+^cells and of the mean fluorescent intensity (MFI) of CD25 among CD4^+^ and CD8^+^T convs when co-cultured with either FL- or BM-MSCs in comparison to T cells alone (Fig. 3). However, this reduction was significantly stronger for FL than BM-MSCs. Furthermore, we evaluated the expression of GITR (TNFRSF18) and TNFR2 (TNFRSF1B), two members of the TNFα receptor superfamily that are upregulated in activated T cells [80–82]. Immediately after 1 day, although we observed a dramatic decrease in the percentage of GITR^+^ cells among CD4+ and CD8+T convs in both MSC groups in comparison to T cells alone, this was more significant for T cells co-cultured with FL-MSCs. MFI of GITR on CD4^+^T and CD8^+^T convs were equally reduced after their co-culture with both MSC groups (Fig. 3). We also observed a dramatic decrease in the percentage of TNFR2^+^cells among CD4^+^ and CD8^+^T convs in both MSC groups in comparison to T cells alone. However, not only was the MFI of TNFR2 not down-modulated much on CD8^+^T cells in the FL-MSC group compared to BM-MSCs, but also it was significantly increased on CD4^+^T cells in the presence of FL-MSCs- (Fig. 3). ICOS co-stimulatory receptor is crucial for T cell activation and proliferation [83]. Twenty-four hours later, we observed a significant decrease in both percentage and MFI of ICOS^+^CD4^+^ and ICOS^+^CD8^+^T cells which was remarkably more down-modulated in FL-MSCs compared to the BM-MSC group (Fig. 3). All the above mentioned activation markers were also quantified after 3 days of co-culture with both MSC groups. Once again, we have observed a more significant down-modulation in percentage of expression of CD25 and GITR in the FL-MSC group compared to BM-MSCs. ICOS was uniquely decreased by FL-MSCs and no significant difference was observed regarding TNFR2 expression level (Supplementary Figure 3). These results indicate that CD4^+^T conv activation profile starts to decrease as soon as day 1 and keeps continuing even 3 days after co-culturing with MSCs. Interestingly, FL-MSCs demonstrated a more powerful and longer-lasting immunomodulatory effect compared to their BM counterpart.

### FL-MSCs have higher capacity to convert Foxp3^−^T convs into Foxp3^+^iT regs than BM-MSCs

**Fig. 4:**
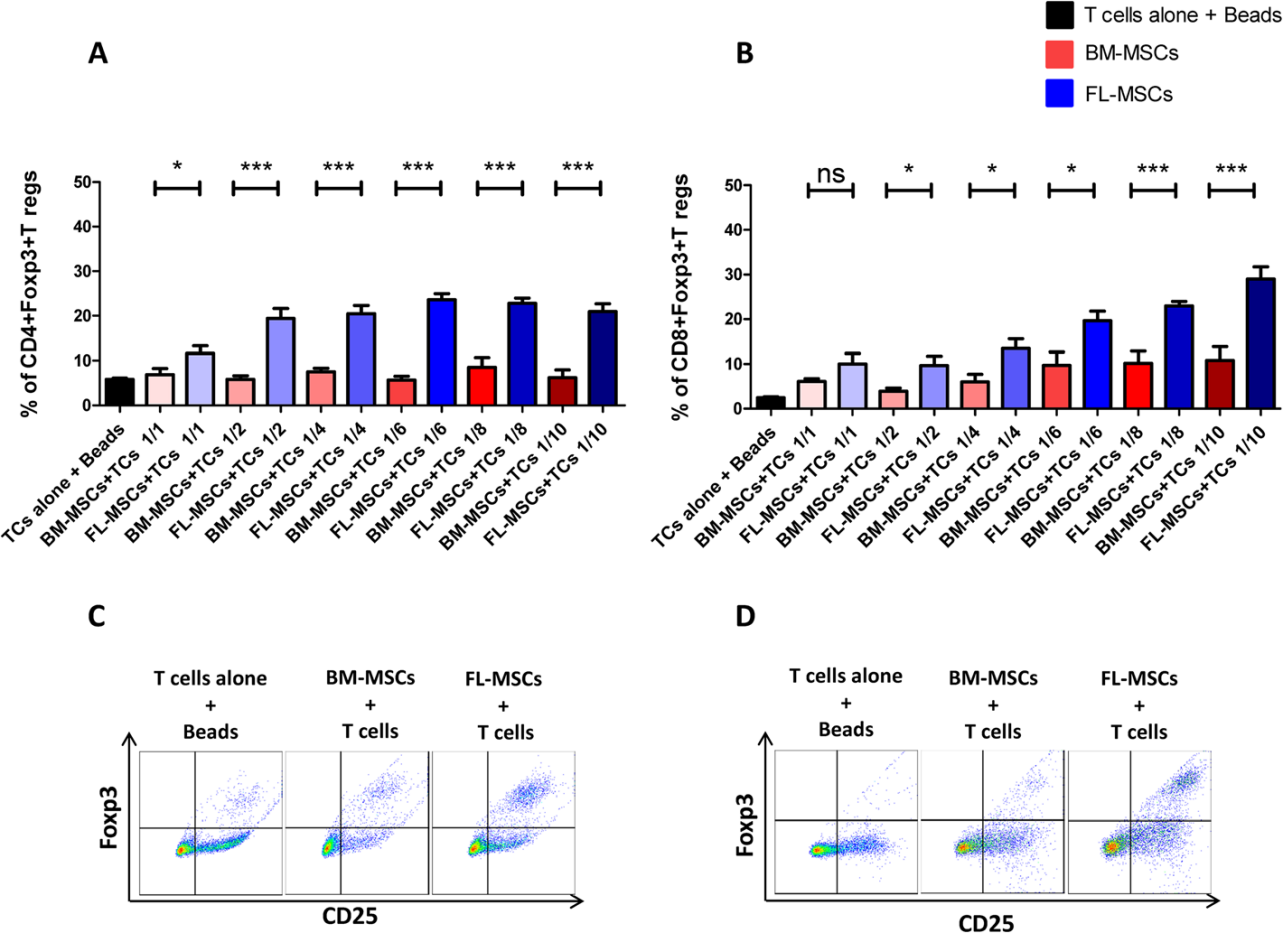
FL-MSCs demonstrate higher Foxp3^+^ T reg induction capacity in comparison to BM-MSCs. CD3/CD28-activated CD3^+^CD25^−^ effector T cells were co-cultured with BM- or FL-MSCs in 6 different ratios. After 4 days, T cells were collected and the expression of CD25 and Foxp3 was determined in CD4^+^ (**a**, **c**) and CD8^+^ (**b**, **d**) cells by flow cytometry measurements. Statistical summary dot-plot graphs showing the percentage of CD25^+^Foxp3^+^ iT regs in CD4^+^ (**a**) and CD8^+^ (**b**) cells. The first black bar represents the percentage of CD25^+^Foxp3^+^ cells in the activated T cells control group (*n* = 18 for CD4 and *n* = 12 for CD8 group). Further bars depict the percentage of CD25^+^Foxp3^+^ cells in activated T cells co-culture with either BM-MSCs (red) or FL-MSCs (blue) (*n* = 13 for CD4 and *n* = 9 for CD8 conditions). Representative FACS dot plots of CD25^+^Foxp3^+^ iT reg populations in activated T cells control group or in activated T cells co-cultures with either BM-MSCs or FL-MSCs (**c**, **d**). Cells were first gated on CD4^+^ (represented in **c**) or CD8^+^ (represented in **d**) cells and then the percentage of CD25^+^Foxp3^+^ double positive cells were determined. The flow cytometry representatives of T reg induction assay were selected from 1:6 MSC to T cell ratio. Data are represented as mean value ± SEM collected from 3 different experiments. One-way ANOVA analysis was performed to generate *P* values. ns, non-significant; Beads, anti-CD3 and anti-CD28 activation beads; TCs, T cells; T regs, regulatory T cells. *P < .05, **P < .01, ***P < .001, ****P < .0001

Because fetal secondary lymphoid organs have an increased frequency of T regs compared to any other period of development [72, 73], we hypothesized that FL-MSCs could enhance a more efficient conversion of CD3^+^CD25^−^T cells to CD4^+^CD25^+^Foxp3^+^ and CD8^+^CD25^+^Foxp3^+^iT regs than adult BM-MSCs. To test this, freshly isolated murine CD3^+^CD25^−^ T cells were activated with anti-CD3/CD28 beads and co-cultured in 6 different MSC to T cell ratios in the presence of either FL- or BM-MSCs. CD25^+^cells were depleted from starting T cells to eliminate activated T cells and unspecific expansion of natural CD25^+^ T regs. Four days later, cells in suspension were collected and analyzed by FACS for the presence of CD4^+^CD25^+^Foxp3^+^ and CD8^+^CD25^+^Foxp3^+^iT regs. The percentage of iT regs was compared to that of activated CD4^+^ or CD8^+^T cells alone (untreated control). For all MSC to T cell ratios, the induction of CD4^+^iT regs (Fig. 4a, c) or CD8^+i^T regs (Fig. 4b, d) were significantly higher in the FL-MSC-treated group than in the BM-MSC one. In the FL-MSCs group Foxp3 induction was dose dependent and reached a maximum at ratios of 1:6 and 1:10 for CD4^+^ iT regs and CD8^+^iT regs, respectively (Fig. 4a, b). In contrast, the increase in CD4^+^ iT regs and CD8^+^ iT regs in the BM-MSC-treated group was lower and steady and did not increase in a dose-dependent manner (Fig. 4a, b).

All together, these observations demonstrated that FL-MSCs induced more strongly the production of CD4^+^CD25^+^Foxp3^+^ and CD8^+^CD25^+^Foxp3^+^ iT regs from T convs than BM-MSCs.

### FL-MSCs induced iT regs display a more active phenotype than those induced by BM-MSCs

**Fig. 5:**
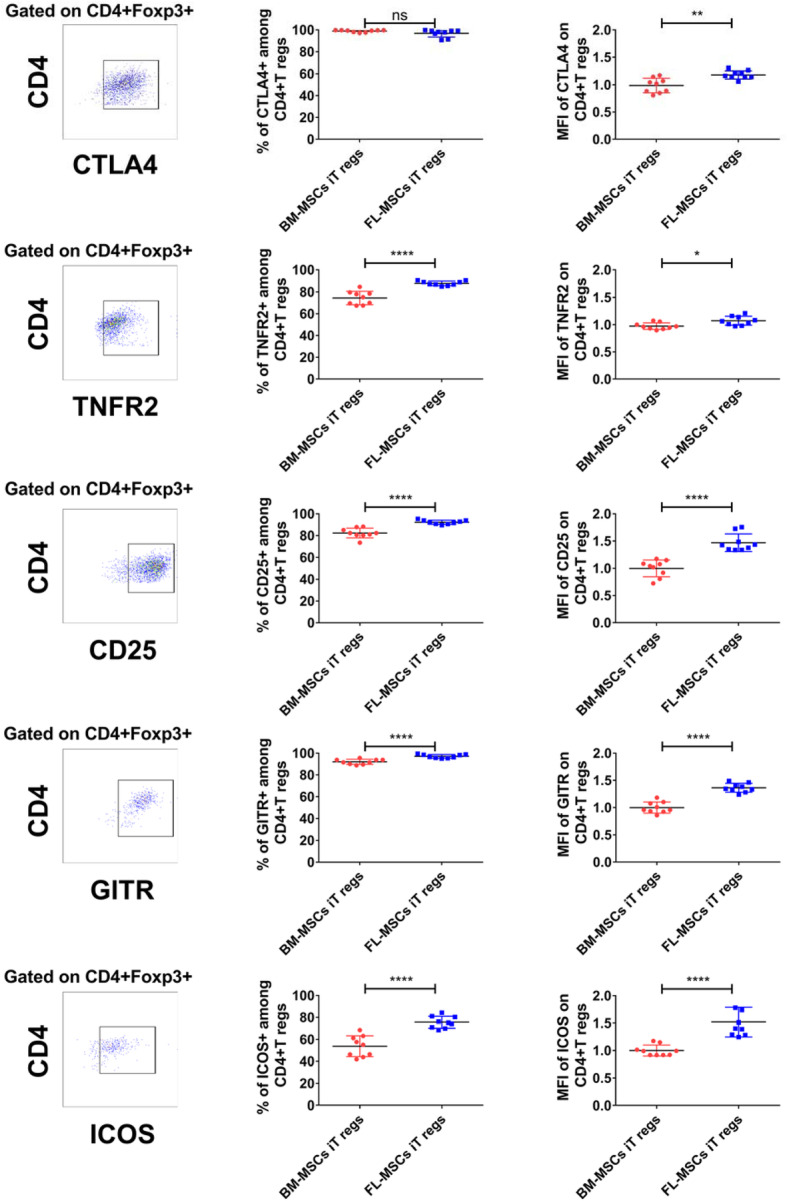
FL-MSCs are able to induce T regs with more activated phenotype in comparison to BM-MSCs. Activated CD3^+^ CD25^−^ effector T cells were co-cultured with BM- or FL-MSCs in a fixed 1:5 MSC to T cell ratio. After 4 days, T cells were collected and the expression of different activated markers (CD25, GITR, TNFR2, and ICOS) was determined in CD4^+^Foxp3^+^ T regs by flow cytometry measurements. Representative flow cytometry dot plots show the percentage of CD25, GITR, TNFR2, and ICOS, within CD4^+^Foxp3^+^ T regs from the FL-MSC group (left panel). After delimitating the lymphocyte region by a forward-scatter-area (FSC-A) versus side-scatter area (SSC-A) plot, a CD4 versus Foxp3 plot was used to gate on CD4^+^Foxp3^+^ T regs. Frames defined the positive subpopulations for each marker analysis in the CD4^+^Foxp3^+^ population. Statistical summary dot-plot graphs showing the percentage or the MFI value of each marker analyzed in CD4^+^Foxp3^−+^ iT regs (right panel). Red dots stand for BM-MSCs iT regs (*n* = 9) and blues stand for FL-MSCs iT regs conditions (*n* = 9). All data are collected from 3 different experiments. MFI values have been normalized with BM-MSCs iT regs group. For each group of values, horizontal lines represent mean value ± SEM. Unpaired Student *t* test analysis was performed to generate *P* values. ns, non-significant; iTregs, induced regulatory T cells. *P < .05, **P < .01, ***P < .001, ****P < .0001

Although FL-MSCs give rise to more iT regs than BM-MSCs, both were able to induce Foxp3 expression. To better define iT regs induced in the presence of FL and BM-MSC, we characterized them and compared their activation/suppressive profile by FACS using different activation/suppressive markers, such as CTLA4, GITR, and ICOS [84–86]. We focused our analysis on commonly accepted CD4^+^Foxp3^+^iT regs. For that purpose, FL and BM-MSCs were co-cultured with anti-CD3/CD28 activated murine CD3^+^CD25^−^T cells at a fixed MSC to T cell ratio (1:5). After 4 days (equal to T reg induction experiments), all cell in suspension were harvested and analyzed by FACS. As anticipated, both FL-MSCs and BM-MSC-derived Foxp3^+^CD4^+^ iT reg populations were highly positive for CTLA4. However, the MFI of this marker was significantly higher in FL-MSCs than BM-MSC-derived Foxp3^+^CD4^+^iT regs (Fig. 5). We and others have shown that one of the most important regulators of T regs activity is TNFR2 which is directly related to their activation and immunosuppressive function [82, 87–89]. Interestingly, we found that both the percentage and expression level of TNFR2 was significantly higher on Foxp3^+^CD4^+^ iT regs after co-culture with FL-MSCs than with BM-MSCs (Fig. 5). The same results were observed for CD25, GITR, and ICOS markers demonstrating a higher global activation of iT regs derived from FL-MSCs than BM-MSCs (Fig. 5).

### FL-MSC-derived iT regs have higher capacity to suppress activated conventional T cells than BM-MSC-derived iT regs

**Fig. 6:**
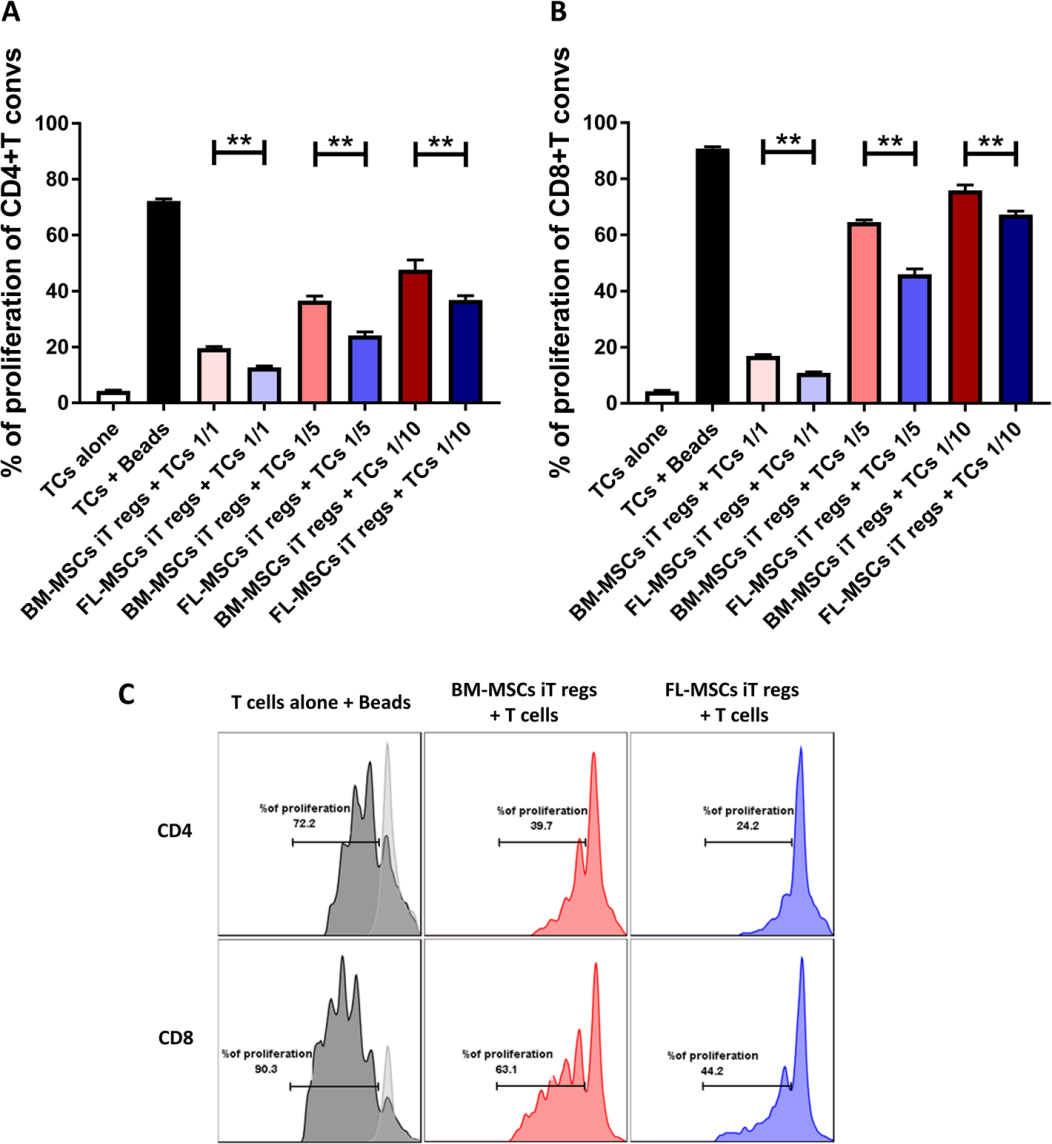
CD4^+^T regs induced from FL-MSCs are more immunosuppressive than those induced from BM-MSCs. Activated CD3^+^CD25^−^ effector T cells were co-cultured with BM- or FL-MSCs in a fixed 1:5 MSC to T cell ratio. After 4 days, CD4^+^CD25^+^ iT regs were selected and co-cultured with CFSE-labeled, CD3/CD28-activated CD3^+^CD25^−^ responder T cells for another 3 days. Then, the proliferation of CD4^+^ (**a**) and CD8^+^ (**b**) T cells was measured by flow cytometry based on CFSE dilution. Each histogram bar represents the percent of dividing cells. The first white bar represents the unstimulated T cells group (*n* = 6), while the second black bar represents the activated T cells group (*n* = 6). T cells co-cultured with BM-MSCs iT regs are depicted in red (*n* = 6) and their FL counterparts in blue (*n* = 6). **c** Representative flow cytometry histogram of proliferation assay at 1:5 MSC to T cell ratio. Non-stimulated T cells are depicted in light gray and CD3/CD28 stimulated T cells are in dark gray. T cells co-cultured with BM-MSCs iT regs are depicted in red and their FL counterparts are depicted in blue. Each histogram bar represents the percent of dividing cells. Data are represented as mean value ± SEM collected from 2 different experiments. One-way ANOVA analysis was performed to generate *P* values. ns, non-significant; TCs, T cells; iT regs, induced regulatory T cells; T convs, conventional T cells; TCs, T cells; iT regs, induced regulatory T cells; Beads, anti-CD3 and anti-CD28 activation beads. *P < .05, **P < .01, ***P < .001, ****P < .0001

Since we observed a higher activation profile in iT regs induced from FL-MSCs compared to BM-MSCs, we sought to confirm that those iT regs were truly able to suppress activated responder T cells in a more efficient manner. To test this, in vitro mixed lymphocyte reaction (MLR) assay was used to determine their suppressive capacity. Activated murine CD3^+^CD25^−^T cells were co-cultured with FL and BM-MSCs for 4 days. Then, CD4^+^CD25^+^iT regs generated in those co-cultures were isolated and co-cultured with freshly isolated, CFSE-labeled, activated murine CD3^+^CD25^−^T cells in 3 different iT reg to T cell ratios (1:1, 1:5, and 1:10). After 3 days of co-culture, the proliferative capacity of CD4^+^ and CD8^+^ T cells were analyzed by flow cytometry. We showed a clear dose-dependent capacity of either FL- or BM-MSC-derived iT regs to inhibit the proliferation of activated CD4^+^ (Fig. 6a, c) or CD8^+^ responder T cells (Fig. 6b, c). Very interestingly, FL-MSC-derived iT regs, at all ratios examined, were more efficient to inhibit either CD4^+^ or CD8^+^T cell proliferation than BM-MSC-derived iT regs. For both FL- and BM-MSC-derived iT regs the highest suppression rate observed for CD4^+^ responder T cells was reached at 1:1 ratio (19.58% and 12.7% of proliferation respectively) and decrease to be the lowest at 1:10 ratio (47.67% and 36.85% of proliferation, respectively) (Fig. 6a). Accordingly, the same results were observed for CD8^+^ responder T cells with the highest suppressive effect at 1:1 ratio (16.97% and 10.85% of proliferation, respectively) and the lowest suppressive effect at 1:10 ratio (75.87% and 67.22% of proliferation, respectively) (Fig. 6b).

We thus demonstrated for the first time that CD4^+^CD25^+^Foxp3^+^iT regs generated during the co-culture with the FL-MSCs have a remarkably higher capacity to suppress murine responder CD3^+^CD25^−^T cells than those generated during co-culture with BM-MSCs.

## Discussion

MSCs are multipotent cells that are being clinically exploited as a new therapeutic for treating a variety of immune-mediated diseases [17–19, 21, 44]. However, frequently used BM derived MSCs are short-living and cannot assure long-lasting immunoregulatory function both in vitro and in vivo [90]. Consequently, several groups including ours have isolated MSCs from other sources with the hope that these cells could have prolonged lifespan and exert longer-lasting immunoregulatory properties [59–62]. Among these alternative sources of MSCs, FL-MSCs seem to be one of the most promising due to their proliferative and differentiation capacity, their low immunogenicity, and their immunomodulatory properties [63, 64, 66, 91]. We and others demonstrated that even though adult BM and FL-MSCs exhibit similar morphology and phenotypes, FL-MSCs demonstrate a faster growing kinetics, a higher number of cells produced over the same period of time, and a longer inhibition of NK and T cell proliferation compared to BM-MSCs [63, 65, 67].

In this study, to go further in our investigations, we have isolated and compared MSCs from FL and adult BM sources to check which one displays the most efficient immunosuppressive effects on CD3/CD28-stimulated CD3^+^CD25^−^ murine T convs either directly or indirectly through T reg induction. Before widespread clinical application of human MSCs, it is required to perform a variety of biological validation tests in murine models. Therefore, we chose to perform our investigations by studying the interaction between human MSCs and mice T cells in order to further test their immunological properties in xenogeneic mice model. Such human MSCs/murine LTs in vitro studies are better mimicking in vivo studies and help to better understand the reaction of the mouse immune system especially conventional T cells against xenogeneic human MSCs.

We first confirmed that although FL and BM-MSCs share several common characteristics, including a spindle-shaped fibroblast-like morphology, phenotype, and differentiation capacities, FL-MSCs bore an outstanding ex vivo expansion ability compared to adult BM-MSCs. Even if both cell types expressed typical MSC markers, we observed a higher expression of ICAM-1 in FL-MSCs and conversely a lower VCAM-1 expression in BM-MSCs. Such differences were already observed between FL and adult BM-MSCs [92]. Whether they play an important role in MSC-mediated immunosuppression still remains to be investigated. We also observed a higher expression of CD146 in FL-MSCs than in BM-MSCs. These results match with a study showing that CD146^+^ BM-MSCs showed greater immunomodulatory functions upon inflammatory priming compared to CD146^−^ BM-MSCs [93]. We also noticed that FL-MSCs contained both CD271^bright^ and CD271^low^ cells, while adult BM-MSCs cultures contained only CD271^low^ cells. This finding is consistent with previous studies demonstrating that CD271^high^ cells have a much higher clonogenic capacity than CD271^low^ cells [76–78] and with our data showing that when seeded in a strictly identical initial number, FL-MSCs proliferate more than BM-MSCs with an effect discernible from day 2.

To investigate the immunomodulatory effects of FL- or BM-MSCs towards activated murine T cells we then performed co-cultures assays. Addition of both FL and BM-MSCs to CD3/CD28-activated CD4^+^CD25^−^ and CD8^+^CD25^−^ T cells reduced their proliferation in a dose-dependent manner. Interestingly, this immunosuppression was significantly higher when FL-MSCs were used compared to BM-MSCs. We then measured the ability of FL and BM-MSCs to modulate conventional murine T cell activation profile by quantifying the expression of CD25, GITR, ICOS, and TNFR2 markers. While both MSC types were able to down-modulate CD4^+^ Foxp3^−^ and CD8^+^ Foxp3^−^T cell activation rapidly after 1 day, this immunomodulatory effect was remarkably stronger with FL than BM-MSCs. Moreover, our results revealed a more effective down-modulation of different T cell activation markers by FL-MSCs even 3 days after their co-culture, meaning that FL-MSCs could potentially render a longer-lasting immunomodulation in comparison to BM-MSCs. Due to the MSCs’ property to reach rapidly the confluency, which restricts the windows of analysis to few days, further in vivo experiments are necessary to evaluate properly the long-term immunosuppressive effect of FL-MSCs.

Interestingly, the increase in TNFR2 expression on T cells in the FL-MSC group could reflect the conversion of T cells towards a more anti-inflammatory phenotype. We and others have already showed that many immunosuppressive cells including T regs, MSCs, and recently endothelial cells derived from circulating endothelial progenitors are expressing TNFR2. This expression is directly related to their immunomodulatory functions [82, 86, 89, 94, 95]. Moreover, the elevated expression of TNFR2 activation marker has been directly correlated to increase IL-10 and TGFβ anti-inflammatory cytokine production [96, 97].

We next checked if FL-MSCs could also exert their immunosuppressive effect indirectly through induction of T regs, as already demonstrated for BM-MSCs [33, 34, 98]. When CD3/CD28-activated CD3^+^CD25^−^ murine T cells were co-cultured in the presence of either FL- or BM-MSCs, we observed CD4^+^CD25^+^Foxp3^+^ and CD8^+^ CD25^+^Foxp3^+^ T regs induction with both MSC types. However, very interestingly, in all conditions tested, FL-MSCs induced a higher percentage of T regs than BM-MSCs.

To better define the phenotypic characteristic of the T reg population induced in the presence of FL- or BM-MSCs, we showed that CTLA4, ICOS, GITR, and CD25, markers commonly used to evaluate T reg activation [84, 85], were higher expressed in the CD4^+^Foxp3^+^ T reg population induced by FL than BM-MSCs. We also checked the expression of TNFR2, one of the most important regulators of T regs activity, that has been shown by us and others to be directly related to their activation and immunosuppressive function [82, 87, 88]. We found that the percentage and the expression level of TNFR2 was significantly higher on CD4^+^Foxp3^+^ iT regs induced by FL-MSCs than with BM-MSCs, reflecting a global activation of the T reg induced population.

Finally, CD4^+^CD25^+^ T reg populations acquired from cultures in the presence of the FL-MSCs or BM-MSCs were then assayed in a secondary MLR, to investigate their immune-modulatory effects on activated murine T cells. While both T reg populations induced from FL- or BM-MSCs were able to inhibit the proliferation of activated responder T cells, FL-iT reg population were significantly more efficient to inhibit CD4^+^ or CD8^+^ T cell proliferation.

Altogether, these results demonstrated that FL-MSCs affected much more murine T cell proliferation and modulate them towards less active phenotypes than adult BM-MSCs. In addition to their substantial suppressive effect, FL-MSCs promoted more effectively the transformation of CD3/CD28-activated CD3^+^CD25^−^ murine T cells into active CD4^+^CD25^+^Foxp3^+^ or CD8^+^CD25^+^Foxp3^+^ T regs and also to a more functional CD4^+^CD25^+^Foxp3^+^ T regs. These results highlight the immunosuppressive activity of FL-MSCs on T cells and show for the first time that one of the main immune-regulatory mechanisms of FL-MSCs is through T reg induction.

To reinforce, validate, and expand the message of this study, we have performed complementary experiments that measure the immunosuppressive effect of human FL and BM-MSCs against human T cells derived from adult peripheral blood (Supplementary Figure 4). We demonstrated that using such a human-human combination leads to the same observations that were acquired from the human-mouse combination and confirmed data already reported in the literature [63, 65].

The physiological more efficient growing advantage of FL-MSCs could potentially explain why FL-MSCs show a higher capacity than BM-MSCs to suppress and modulate effector T cells (more cells, more immunosuppressive effects). This more powerful immunomodulatory effect could be through either cell to cell contact or via the secretion of anti-inflammatory mediators. The increase in anti-inflammatory cytokines production such as IL-10 and TGf-β by FL-MSCs could also cause the induction of more CD4^+^Foxp3^+^ and CD8^+^Foxp3^+^ T regs that indirectly leads to more immunosuppression of effector T cells. The other possibility is that when compared to a strictly similar number of BM-MSCs, FL-MSCs might have a stronger capacity to directly suppress effector T cells and/or to induce T regs (one cell, more immunosuppressive capacity).

T reg induction by fetal MSCs not only highlights the existing relation between MSCs and T regs [31] but also the role played by fetal T regs in tolerance to maternal antigens in utero [99–103]. One of the main challenges of early life is to keep the equilibrium between producing appropriate and robust immune responses to pathogens and developing tolerance to self and harmless antigens. Several pathways restricting T cell reactivity during early life have been identified which are either specific or enhanced compared to adult life ones. In particular, an increased frequency of T regs is observed in peripheral tissues and blood during fetal life and the function of fetal T regs is strengthened relative to T regs derived from adult tissues [68–71]. This abundance of T regs is not reflected in the thymus of equivalent gestational age, where the frequency of CD25^+^Foxp3^+^ among CD4^+^ thymocytes is comparable to the infant thymus [68]. This suggests that a significant portion of fetal T regs are derived from expansion of natural T regs or are generated from conventional CD4^+^Foxp3^−^ T cells in response to antigen. In agreement with this hypothesis, authors demonstrated that when fetal naïve CD4^+^ T cells are isolated and stimulated with alloantigens, they exhibit a strong predisposition to differentiate into T regs, as compared to adult naïve CD4^+^ T cells, thereby biasing the immune system towards tolerance [72, 73]. Hematopoiesis during fetal development takes place in waves, each one generating separate T cell populations that may coexist for a period of time [73]. As the source of hematopoiesis switches from the FL to the fetal BM, the effector T cell/regulatory T cell ratio progressively shifts towards that found in adults [104–106]. Interplay between FL-MSCs and T cells during fetal life may be a mechanism by which fetal naïve CD4^+^ T cells preferentially differentiate into T regs. Whether FL-MSCs influence T reg conversion and how this interaction contributes to sustained T reg expansion during fetal life warrant further investigation. These results are also in favor of the development of new tools and strategies based on the use of FL-MSCs cells and their derivatives for the induction of immune tolerance. The implications of these findings for clinical application could be especially important if the fetal T reg population induced by FL-MSCs is found to promote tolerance not only to self and non-inherited maternal antigens, but also to foreign antigens encountered. Further insights into the fetal liver-derived T regs, including their unique immunomodulatory properties, could result in novel strategies to regulate alloimmune and autoimmune responses.

## Conclusion

Here, we demonstrated that although both FL and BM-MSC exhibit similar phenotype profile and differentiation capacity, FL-MSCs have significantly higher capacity to suppress both CD4^+^ and CD8^+^ human and murine T cell proliferation and to modulate murine T cells towards less active phenotypes than adult BM-MSCs. Moreover, FL-MSCs enhance more efficiently the conversion of murine T cells to T regs than BM-MSCs. These results suggest that the FL microenvironment could play a role in controlling immune responses during development, either directly by immunomodulation fetal T cells or indirectly by generating iT regs from T convs. Beside the fundamental importance of this study, the influences of MSCs on T regs could represent an important element of the therapeutic effects of MSCs for the treatment of immune-related disorders and transplantations. In a combinatorial approach, MSCs and T regs could synergize each other’s immunoregulatory functions and exert advantageous complementary effects.

## Supplementary Information

**Additional file 1: Supplementary Figure 1.** FL-MSCs contained both CD271^bright^ and CD271^low^ cells, while adult BM –MSCs contained only CD271^low^ cells. FL and BM MSCs cultured at passage 4, were stained with APC-anti-CD271 Mab. Representative flow cytometry histograms and dot plots show the relative expression of CD271 by FL and BM-MSCs. Numbers indicate the percentage of CD271^bright^ cells in the corresponding histogram bars and quadrants. Data are representative of 3 independent experiments (*n* = 3).

**Additional file 2: Supplementary Figure 2.** In-vitro osteogenic and adipogenic differentiation capacity of FL-MSCs compared to BM-MSCs. FL (passage 4) and BM (passage 4)-MSCs were cultured with or without inductive media to induce osteogenic or adipogenic cell differentiation. Representative images of osteogenic and adipogenic differentiation detected by Alizarin Red S and Oil Red O staining, respectively. Data are representative of 2 independent experiments (*n* = 6). Scale bar indicates 100 μm.

**Additional file 3: Supplementary Figure 3.** Long-lasting down-modulation of CD4^+^ and CD8^+^ T convs by FL-MSCs compare to BM-MSCs CD3/CD28 activated CD3^+^CD25^−^ effector T cells were co-cultured with BM-MSCs or FL-MSCs in a fixed 1:5 MSC to T cell ratio. After 3 day, T cells were collected and T cell activation markers (CD25, GITR, ICOS and TNFR2) were analyzed by flow cytometry. Statistical summary dot-plot graphs showing the percentage of each marker analyzed in CD4^+^Foxp3^−^ (A) or CD8^+^Foxp3^−^ (B) T convs. Each dot represents a measured value collected from 2 different experiments (*n* = 12 for T cells + Beads group (black) and *n* = 9 for BM-MSCs + T cells (red) and FL-MSCs + T cells (blue) groups). For each group of values, horizontal lines represent mean value ± SEM. One way ANOVA analysis was performed to generate *P* values. ns: non-significant, *P < .05, **P < .01, ***P < .001, ****P < .0001. Beads: Anti-CD3 and anti-CD28 activation Beads; T convs: conventional T cells.

**Additional file 4: Supplemental Figure 4.** FL-MSCs are more immunosuppressive against human HLA mismatched T cells than BM-MSCs. CFSE labeled, CD3/CD28 activated CD3^+^CD25^−^ human effector T cells were co-cultured with FL-MSCs or BM-MSCs in 6 different MSC to T cell ratios. After 3 days, proliferation of CD4^+^ (A) and CD8^+^ T cells (B) was measured by flow cytometry based on CFSE dilution. Each bar represents the percent of dividing cells. The first bar represents the unstimulated T cells alone (*n* = 4), the second bar represents the CD3/CD28-stimulated T cells alone (n = 4). Further bars depict T cells co-cultured with either BM-MSCs in red (n = 4) or FL-MSCs in blue (n = 4). Data are represented as mean value ± SEM. One way ANOVA analysis was performed to generate P values. ns: non-significant, *P < .05, **P < .01, ***P < .001, ****P < .0001. Beads: Anti-CD3 and anti-CD28 activation Beads; TCs: T cells; T convs: conventional T cells.

**Additional file 5: Supplemental Table S1.** Monoclonal antibodies used in this study.

## Data Availability

The datasets used and/or analyzed during the current study are available from the corresponding author on reasonable request.

## Abbreviations

BM: Bone marrow
FL: Fetal liver
GITR: Glucocorticoid-induced TNF receptor family related gene
ICOS: Inducible co-stimulatory molecule
iT regs: Induced regulatory T cells
MFI: Mean fluorescence intensity
MHC: Major histocompatibility complex
MSCs: Mesenchymal stem cells
T conv: Conventional T cell
TNFR2: Tumor necrosis factor receptor 2
