# Supplementary figures and images for: Human fetal liver MSCs are more effective than adult bone marrow MSCs for their immunosuppressive, immunomodulatory, and Foxp3+ T reg induction capacity
Source: Stem Cell Res Ther. 2021 Feb 17;12:138. doi: 10.1186/s13287-021-02176-1 (PMC7888159; doi:10.1186/s13287-021-02176-1)

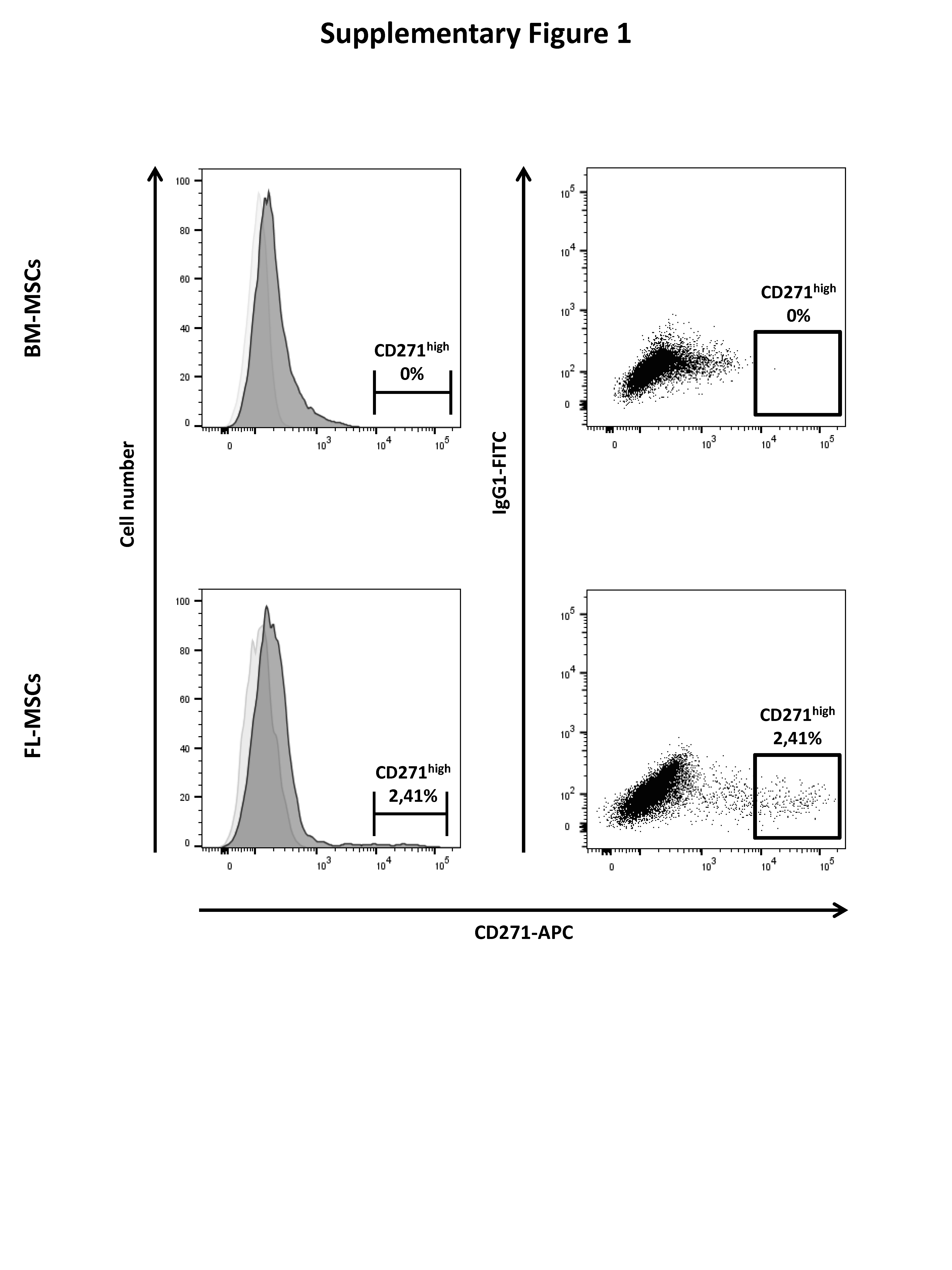

Supplement: Supplementary file 1 — Additional file 1: Supplementary Figure 1. FL-MSCs contained both CD271bright and CD271low cells, while adult BM –MSCs contained only CD271low cells. FL and BM MSCs cultured at passage 4, were stained with APC-anti-CD271 Mab. Representative flow cytometry histograms and dot plots show the relative expression of CD271 by FL and BM-MSCs. Numbers indicate the percentage of CD271bright cells in the corresponding histogram bars and quadrants. Data are representative of 3 independent experiments (n = 3). [file 13287_2021_2176_MOESM1_ESM.tiff]

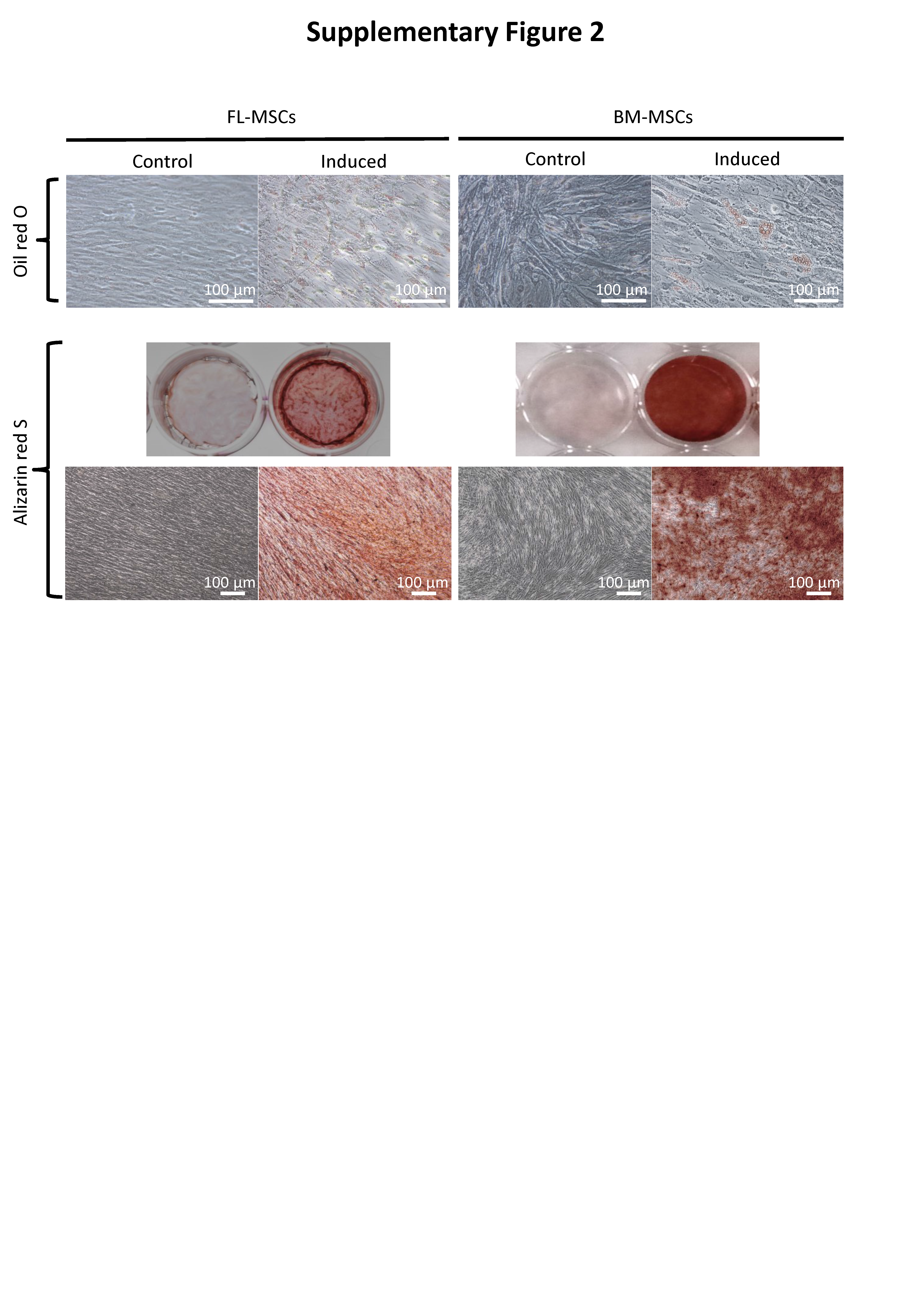

Supplement: Supplementary file 2 — Additional file 2: Supplementary Figure 2. In-vitro osteogenic and adipogenic differentiation capacity of FL-MSCs compared to BM-MSCs. FL (passage 4) and BM (passage 4)-MSCs were cultured with or without inductive media to induce osteogenic or adipogenic cell differentiation. Representative images of osteogenic and adipogenic differentiation detected by Alizarin Red S and Oil Red O staining, respectively. Data are representative of 2 independent experiments (n = 6). Scale bar indicates 100 μm. [file 13287_2021_2176_MOESM2_ESM.tiff]

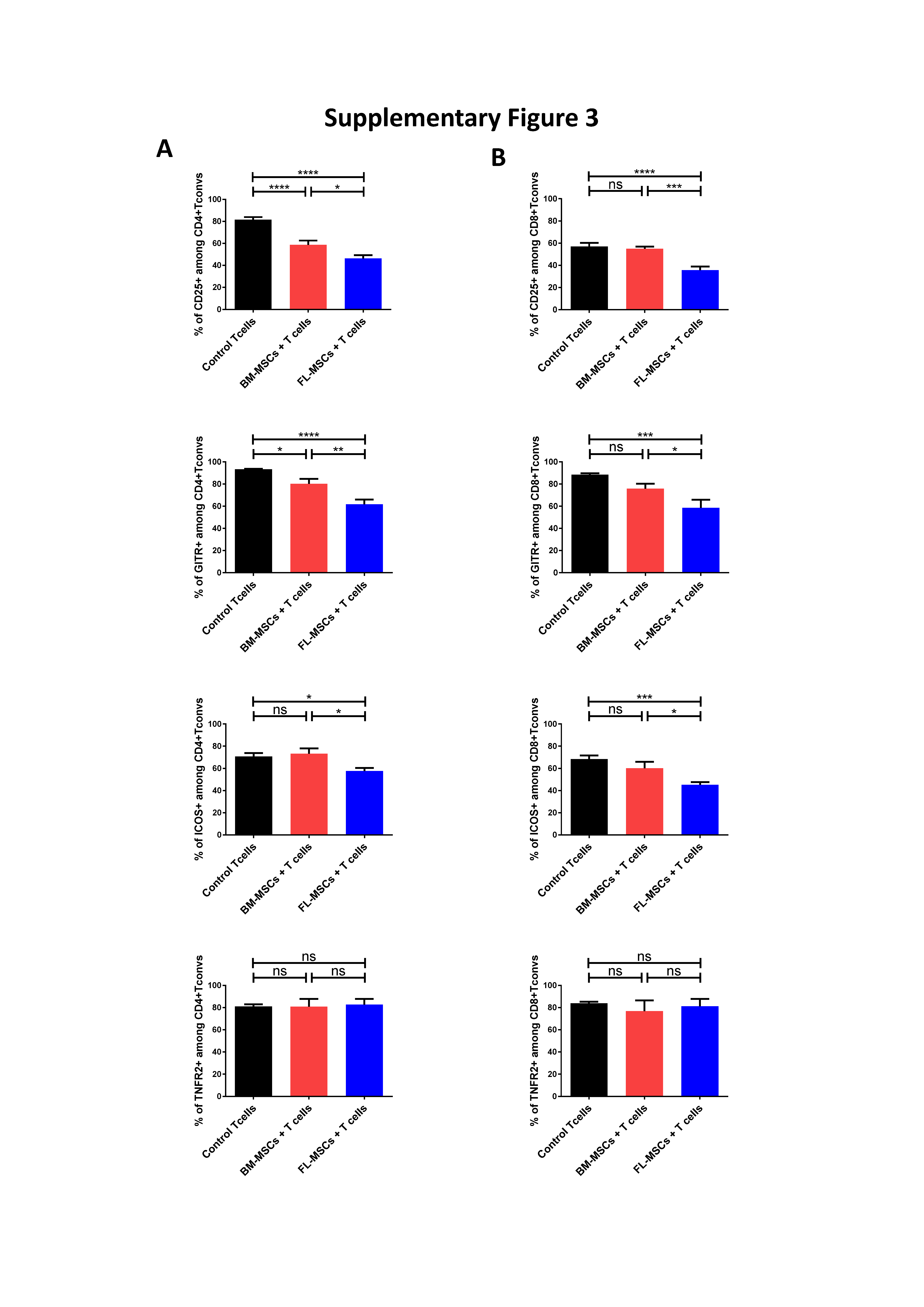

Supplement: Supplementary file 3 — Additional file 3: Supplementary Figure 3. Long-lasting down-modulation of CD4+ and CD8+ T convs by FL-MSCs compare to BM-MSCs CD3/CD28 activated CD3+CD25− effector T cells were co-cultured with BM-MSCs or FL-MSCs in a fixed 1:5 MSC to T cell ratio. After 3 day, T cells were collected and T cell activation markers (CD25, GITR, ICOS and TNFR2) were analyzed by flow cytometry. Statistical summary dot-plot graphs showing the percentage of each marker analyzed in CD4+Foxp3− (A) or CD8+Foxp3− (B) T convs. Each dot represents a measured value collected from 2 different experiments (n = 12 for T cells + Beads group (black) and n = 9 for BM-MSCs + T cells (red) and FL-MSCs + T cells (blue) groups). For each group of values, horizontal lines represent mean value ± SEM. One way ANOVA analysis was performed to generate P values. ns: non-significant, *P < .05, **P < .01, ***P < .001, ****P < .0001. Beads: Anti-CD3 and anti-CD28 activation Beads; T convs: conventional T cells. [file 13287_2021_2176_MOESM3_ESM.tiff]

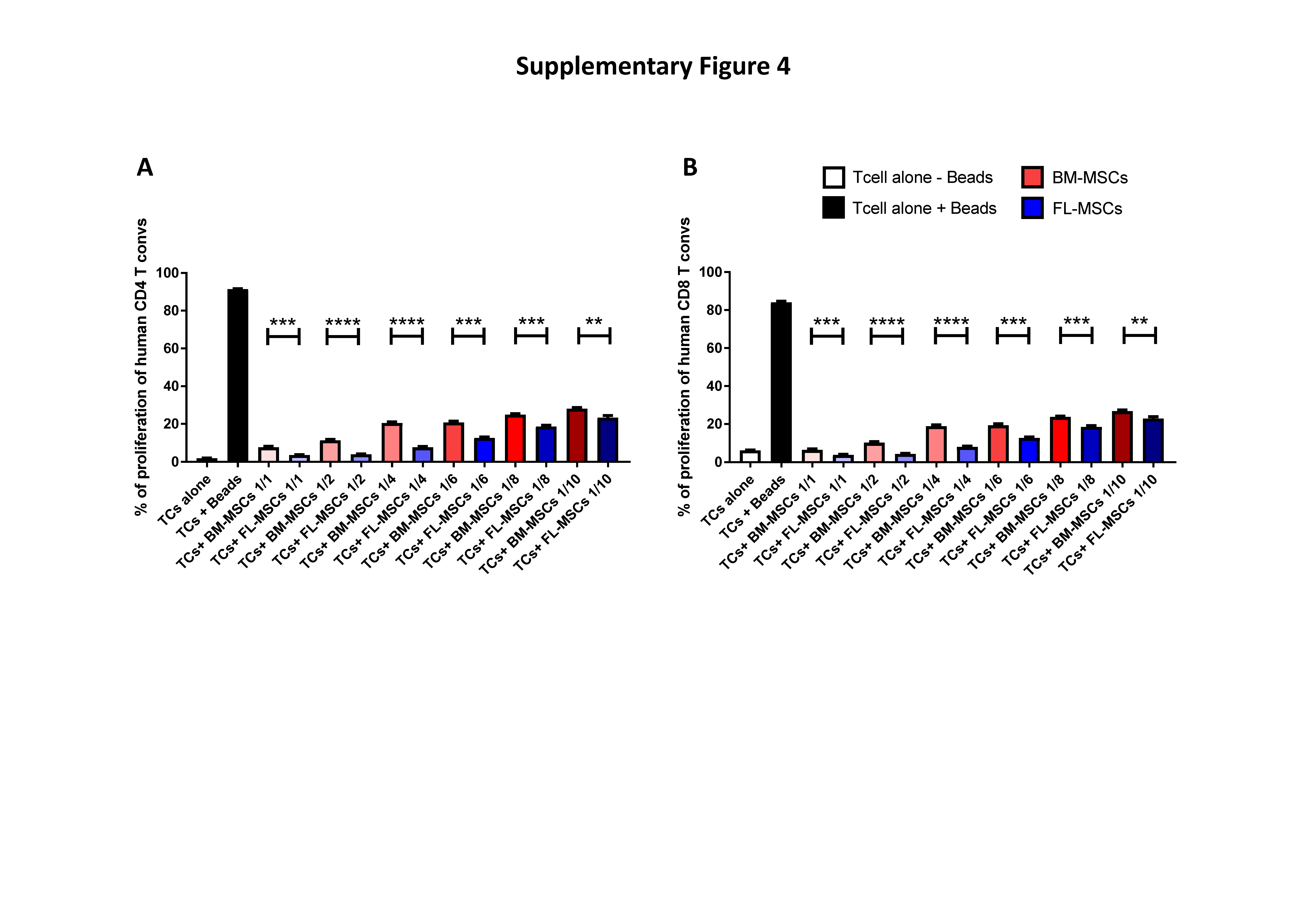

Supplement: Supplementary file 4 — Additional file 4: Supplemental Figure 4. FL-MSCs are more immunosuppressive against human HLA mismatched T cells than BM-MSCs. CFSE labeled, CD3/CD28 activated CD3+CD25− human effector T cells were co-cultured with FL-MSCs or BM-MSCs in 6 different MSC to T cell ratios. After 3 days, proliferation of CD4+ (A) and CD8+ T cells (B) was measured by flow cytometry based on CFSE dilution. Each bar represents the percent of dividing cells. The first bar represents the unstimulated T cells alone (n = 4), the second bar represents the CD3/CD28-stimulated T cells alone (n = 4). Further bars depict T cells co-cultured with either BM-MSCs in red (n = 4) or FL-MSCs in blue (n = 4). Data are represented as mean value ± SEM. One way ANOVA analysis was performed to generate P values. ns: non-significant, *P < .05, **P < .01, ***P < .001, ****P < .0001. Beads: Anti-CD3 and anti-CD28 activation Beads; TCs: T cells; T convs: conventional T cells. [file 13287_2021_2176_MOESM4_ESM.tiff]
