# Supplementary material for: Human fetal liver MSCs are more effective than adult bone marrow MSCs for their immunosuppressive, immunomodulatory, and Foxp3+ T reg induction capacity
Source: Stem Cell Res Ther. 2021 Feb 17;12:138. doi: 10.1186/s13287-021-02176-1 (PMC7888159; doi:10.1186/s13287-021-02176-1)
Supplement: Supplementary file 5 — Additional file 5: Supplemental Table S1. Monoclonal antibodies used in this study. [file 13287_2021_2176_MOESM5_ESM.docx]

**Supplemental Table S1. Monoclonal antibodies used in this study**

| **Specificity** | **Form** | **Isotype** | **Clone** | **Manufacturer** | **Use** |
| --- | --- | --- | --- | --- | --- |
| **Murine CD4** | Vioblue | Human IgG1* | REA604 | Miltenyi | FC |
| **Human CD4** | Vioblue | Human IgG1 | REA623 | Miltenyi | FC |
| **Murine CD8α** | VioBright FITC | Human IgG1* | REA601 | Miltenyi | FC |
| **Murine CD8α** | PE-Vio770 | Rat IgG2a | 53-6.7 | Miltenyi | FC |
| **Human CD8** | PE-Vio770 | Mouse IgG2a | BW135/80 | Miltenyi | FC |
| **Human CD14** | FITC | Mouse IgG2a | RM052 | Beckman Coulter | FC |
| **Murine CD25** | Biotin | Mouse IgM | 7D4 | BD Pharmingen | CS |
| **Murine CD25** | PE-Cy7 | Rat IgG1 | C61 | BD Pharmingen | FC |
| **Human CD25** | Biotin | Human IgG1* | REA570 | Miltenyi | CS |
| **Human CD29** | APC | Mouse IgG1 | TS2/16 | Miltenyi | FC |
| **Human CD34** | APC | Mouse IgG2a | AC136 | Miltenyi | FC |
| **Human CD44** | PE | Mouse IgG2b | G44-26 | BD Pharmingen | FC |
| **Human CD45** | FITC | IgG1 | J33 | Beckman Coulter | FC |
| **Human CD51** | APC | Human IgG1* | EA181 | Miltenyi | FC |
| **Human CD54 (ICAM-1)** | APC | Human IgG1* | REA266 | Miltenyi | FC |
| **Human CD73** | PE | Mouse IgG1 | AD2 | BD Pharmingen | FC |
| **Human CD90** | FITC | Mouse IgG1 | DG3 | Miltenyi | FC |
| **Human CD105 (Endoglin)** | APC | Mouse IgG1 | 43A4E1 | Miltenyi | FC |
| **Human CD106 (VCAM-1)** | PE | Human IgG1* | REA269 | Miltenyi | FC |
| **Murine CD120b (TNF-R2)** | APC | Human IgG1* | REA228 | Milenyi | FC |
| **Human CD144** | PE | Murine IgG1 | TEA1/31 | Beckman Coulter | FC |
| **Human CD146** | APC | Mouse IgG1 | 41-10B2 | Miltenyi | FC |
| **Murine CD152 (CTLA4)** | PE | Hamster IgG1 | UC10-4B9 | Miltenyi | FC |
| **Human CD166 (ALCAM)** | PE | Mouse IgG1 | 3A6 | BD Pharmingen | FC |
| **Human CD271 (LNGFR)** | APC | Human IgG1* | REA844 | Miltenyi | FC |
| **Murine CD278 (ICOS)** | PE-Vio770 | Rat IgG2b | 7E.17G9 | Miltenyi | FC |
| **Murine CD357 (GITR)** | PE | Rat IgG2b | DTA-1 | Miltenyi | FC |
| **Murine FoxP3** | PE-CY5.5 | Rat IgG2a | FJK-16s | eBioscience | FC |
| **Human HLA-ABC** | FITC | Mouse IgG2a | B9.12.1 | Beckman Coulter | FC |
| **Human HLA-DR** | FITC | Mouse IgG2a | G46-6 | BD Pharmingen | FC |
| *Recombinant Human IgG1  Flow cytometry: FC  Cell sorting: CS | | | | | |
